# Surgical nerve wrapping for brachial plexus neuropathy: A systematic review

**DOI:** 10.1016/j.jpra.2025.11.005

**Published:** 2025-11-11

**Authors:** François Thuau, Guillaume Gadbled, Théodore Lahmar, Pierre Perrot, Ugo Lancien

**Affiliations:** a CHU Nantes, Plastic Reconstructive, and Aesthetic Surgery Department, Nantes University, 1 Place Alexis Ricordeau, Nantes, France; b CHU Nantes, Orthopedic and Traumatology Surgery Department, Nantes University, 1 Place Alexis Ricordeau, Nantes, France; c INSERM, UMRS 1229, Laboratory Regenerative Medicine and Skeleton (RMeS), 1 Place Alexis Ricordeau, Nantes, France

**Keywords:** Wrapping, Systematic review, Brachial plexus, Plexopathy, Neurogenic thoracic outlet syndrome

## Abstract

**Introduction:**

Nerve wrapping provides protection, reduces adhesions, and supports regeneration, with proven benefits in median and ulnar neuropathies. Brachial plexus neuropathies—arising from neurogenic thoracic outlet syndrome (NTOS), radiation-induced brachial plexopathy (RIBP), or trauma—pose distinct challenges, and indications for wrapping remain poorly defined. We conducted a systematic review of brachial plexus wrapping techniques to clarify approaches and outcomes.

**Methods:**

PubMed, Scopus, Cochrane Library, and ProQuest were searched following PRISMA guidelines; the protocol was registered on PROSPERO. Risk of bias was assessed with the MINORS tool. Extracted data included patient characteristics, surgical techniques, functional outcomes, complications, and recurrences.

**Results:**

Seventeen studies involving 645 patients (mean age 43.8 years; M/F 1/3.92) were included. The main indications were NTOS (77.1 %) and RIBP (22.6 %). Eleven wrapping techniques were identified: eight different flaps (*n* = 214) from omental, muscular, or adipofascial origins, and three medical devices (*n* = 426). NTOS was mainly treated with synthetic membranes, and RIBP with flaps. Pain reduction was reported in 91.9 % of cases, with complete relief in 62.8 %. For RIBP, motor improvement (52.5 %) and sensory improvement (50 %) were observed, while QuickDASH scores for NTOS improved by a mean of 23.3 points. Comparative studies (*n* = 4) suggest wrapping may outperform neurolysis alone in RIBP.

**Conclusion:**

Brachial plexus wrapping may improve pain and function in selected patients, but current evidence is heterogeneous, retrospective, and at high risk of bias. Standardized, prospective controlled studies are required to determine its role relative to isolated neurolysis.

Level of evidence: III

## Introduction

Nerve wrapping is a well-established surgical technique widely used to promote nerve regeneration. Traditionally, it has been used as a conduit for axonal regrowth following nerve repair, facilitating healing and preventing perineural adhesions. More recently, there has been increasing interest in its therapeutic potential for the management of neuropathies, particularly those of compressive origin.

The rationale behind nerve wrapping is multifaceted and depends on the material employed. It can provide mechanical protection to the nerve, reduce local inflammation, minimize perineural adhesion formation, and even deliver essential trophic support crucial for nerve recovery. This approach has been thoroughly documented for commonly affected peripheral nerves, such as the median and ulnar nerves, as evidenced by studies on revision decompression and nerve wrap for recurrent or persistent compressive neuropathies, and the use of collagen conduits or adipose flap.^1^, ^2^, ^3^, ^4^, ^5^, ^6^, ^7^, ^8^

However, managing neuropathies affecting the brachial plexus presents unique challenges. These neuropathies often result from compression (Neurogenic thoracic outlet syndrome (NTOS)), perineural fibrosis due to multiple surgeries, a history of irradiation, or complex post-traumatic scarring. Despite the promising potential of nerve wrapping in these contexts, specific indications for the brachial plexus remain rare, and the literature is notably sparse. Currently, there is no comprehensive overview of the various nerve wrapping techniques applicable to the brachial plexus.

This systematic review therefore aims to address this gap by synthesizing all available nerve wrapping techniques reported in the literature specifically for brachial plexus wrapping, excluding cases of direct conduit placement after nerve suture. Our objective is to provide a complete understanding of existing surgical approaches and their potential applications in this complex anatomical region.

## Methods

### Study design

This systematic review was conducted in accordance with the PRISMA (Preferred Reporting Items for Systematic Reviews and Meta-Analyses) guidelines.^9^ The study protocol was registered in the PROSPERO database (CRD42025640960) to ensure methodological transparency and adherence to best practices for systematic reviews.

### Search strategy

A comprehensive search was performed in the following databases from their inception to January 2025: PubMed, Scopus, Cochrane Library, and ProQuest Dissertations and Theses Global. The search terms were designed to capture a wide range of relevant studies and were based on the MeSH (Medical Subject Headings) thesaurus. The detailed search strategy used is available in Supplementary Material. The reference lists of all selected articles were also screened to identify additional relevant studies.

### Eligibility criteria

Eligible studies enrolled patients of any age who underwent nerve wrapping for brachial plexus neuropathy and provided a clear description of the surgical technique; only articles written in English or French were considered. We excluded studies in which nerve wrapping was used primarily for nerve repair (for example, wrapping a nerve suture with a vein), as well as letters to the editor without case reports, reviews without original data, animal or cadaveric studies, and conference abstracts.

### Data extraction and management

The selection and data extraction process was performed using Zotero 7.0 software (George Mason University, Virginia, USA). After duplicate removal, titles and abstracts were screened, and irrelevant articles were excluded. Full-text articles were then assessed for eligibility. Most of the selected articles were accessible online. For articles with restricted access, the corresponding authors were contacted directly. Two independent reviewers conducted each stage of the process, and disagreements were resolved by a third reviewer.

Extracted data from each study included: study characteristics (authors, year, study design, sample size); patient demographics (age, sex, etiology); intervention details (type of wrapping material, surgical technique); outcomes (functional recovery, pain improvement, quality of life, complications, follow-up duration).

### Quality assessment

The methodological quality and risk of bias of the included studies were assessed using the MINORS tool (Methodological Index for Non-Randomized Studies).^10^ Two independent reviewers assigned the scores; disagreements were resolved through discussion or consultation with a third reviewer.

### Data synthesis

A narrative synthesis of the results was performed, with data categorized according to patient demographics, type of intervention, and clinical outcomes. Quantitative data were presented in tables, and descriptive statistical analyses were conducted. A meta-analysis was considered unlikely due to the expected heterogeneity of outcome measures. Subgroup analyses were planned to explore differences based on the type of wrapping material used and the etiology of the neuropathy.

## Results

This systematic review identified 17 studies reporting nerve wrapping techniques applied to the brachial plexus, encompassing a total of 645 patients. The analysis revealed two main categories of wrapping materials, vascularized tissue flaps and medical devices, with encouraging outcomes in terms of pain relief and functional improvement.

### Study selection

The literature search identified a total of 3024 references from the following databases: PubMed (*n* = 1050), ProQuest Dissertations and Theses Global (*n* = 542), Scopus (*n* = 1204), and the Cochrane Library (*n* = 228). In addition, six further articles were identified by screening the reference lists of included studies.

**Figure 1:**
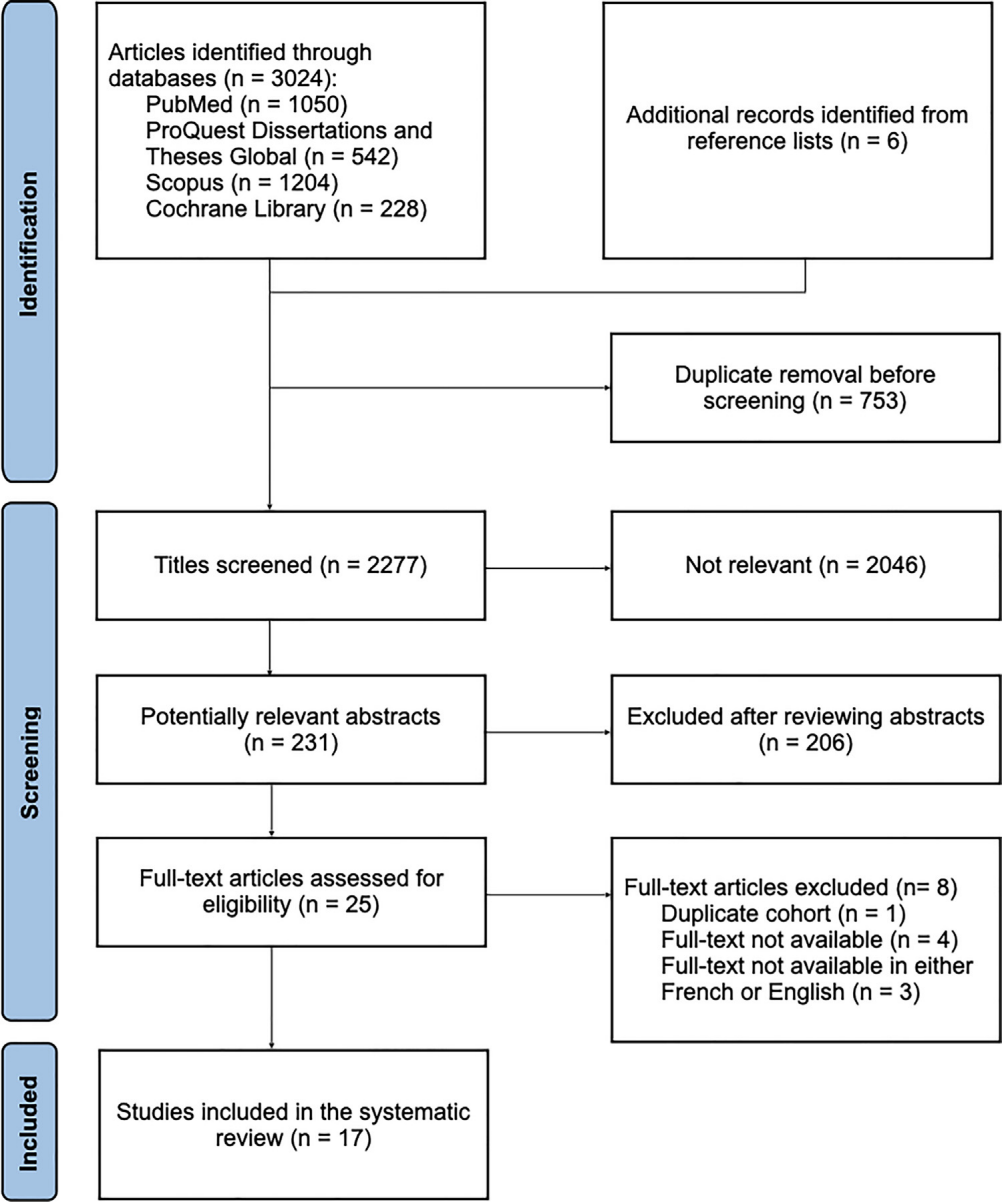
PRISMA flow diagram of study selection.

**Figure 2:**
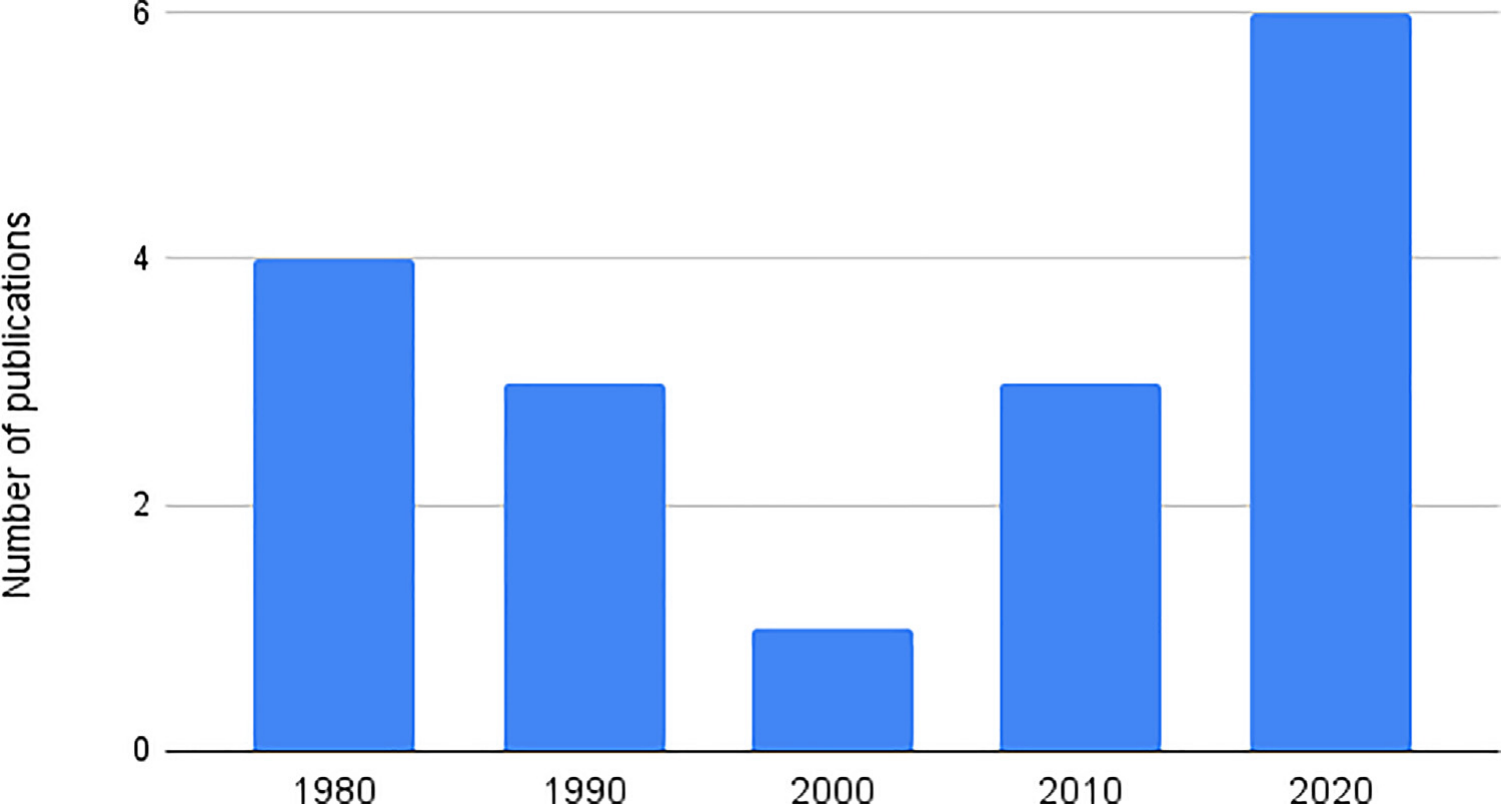
Distribution of included publications by decade.

After removing duplicates (*n* = 753), 2277 references were screened by title. At this stage, 2046 articles were excluded as irrelevant. The remaining 231 abstracts were reviewed, of which 206 were excluded for not meeting the inclusion criteria. A total of 25 full-text articles were assessed. Among these, eight were excluded for the following reasons: full text not available (*n* = 4),^11^, ^12^, ^13^, ^14^ full text available in a language other than French or English (*n* = 3), and duplicate reporting of the same patient cohort (*n* = 1).^15^^,^^16^ Ultimately, 17 studies were included in the systematic review.^16^, ^17^, ^18^, ^19^, ^20^, ^21^, ^22^, ^23^, ^24^, ^25^, ^26^, ^27^, ^28^, ^29^, ^30^, ^31^, ^32^ The PRISMA flow diagram (Figure 1, Figure 2) summarizes the selection process.

### Risk of bias

All included studies had a retrospective design with generally low methodological quality (Table 1). The MINORS tool yielded a mean score of 7.8/16 for non-comparative studies (range: 0–12) and 12.2/24 for comparative studies (range: 11–14). Only four studies (23.5 %) demonstrated intermediate methodological quality, while 13 studies (76.5 %) were considered of poor quality, reflecting a high risk of bias. Strengths included a lost-to-follow-up rate below 5 % (mean score: 1.6/2), an adequate follow-up duration (mean score: 1.5/2), and consecutive patient inclusion in some studies (mean score: 1.2/2). However, major limitations were identified: no study performed a prospective sample size calculation (0/16), outcome assessments were systematically biased (unblinded) (0/16), and comparative statistical analyses were inadequate in the relevant studies. These methodological limitations call for cautious interpretation of the results.

**Table 1:** Risk of bias assessment using the MINORS tool (Methodological Index for Non-Randomized Studies).

### Study and patient characteristics

Our systematic review included 17 studies, comprising 13 case series (two of which were more technical descriptions) and four case reports. Four case series were comparative, comparing brachial plexus wrapping to neurolysis alone or to conservative management.

In total, 645 patients were analyzed, with a mean age of 43.8 years (ranging from 15 to 79 years). These patients underwent 698 surgical procedures, including 640 nerve wrapping procedures.

A marked female predominance was observed (122 men and 478 women; sex ratio 1:3.92), reflecting the main surgical indications. Two main etiologies dominated: NTOS accounted for 77.1 % of cases (*n* = 538), divided into primary forms (*n* = 186) and secondary forms (*n* = 352); radiation-induced brachial plexopathy (RIBP) accounted for 22.6 % of cases (*n* = 158), mostly following radiotherapy for breast cancer or Hodgkin’s lymphoma.

Anecdotal cases were also reported, such as wrapping of a traumatic post-amputation axillary neuroma or scar-related plexus entrapment following osteosynthesis of an acromioclavicular disjunction. It is noteworthy that comparative studies only concerned RIBP, limiting conclusions for other indications.

The mean follow-up duration was 59.8 months (ranging from 1 to 348 months), calculated based on the 13 studies that provided this information. Table 2 summarizes the characteristics of the included studies and patient demographics.

**Table 2:** Demographic characteristics of the patients in each study.

Authors (year)	Country	Design	Number of patients (*n*)	Number of procedures (*n*)	Sex-ratio (M/F)	Mean age (min-max)	Indication (*n*)	Nerve wrapping technique	Number of wrapping procedures (*n*)	Pedicled flap (*n*)	Free flap (*n*)	Comparator (*n*)	Mean follow-up duration, in months (min-max)
Sessions^19^	USA	Case series	29	37	6/23	NR	RNTOS (37)	Scalene fat pad	28	28	-	-	23 (1–84)
Narakas^20^	Switzerland	Case series	40	40	2/38	50.3 (19–73)	RIBP (40)	Omental flap	15	11	4	Conservative treatment (11) Neurolysis alone (14)	52.5 (6–192)
Brunelli and Brunelli^16^	Italy	Case series	37	37	NR	NR	RIBP (37)	Omental flap	31	0	31	Neurolysis alone (6)	NR (6–96)
LeQuang^21^	France	Case series	57	57	1/56	57 (18–79)	RIBP (57)	Omental Flap Latissimus dorsi flap Pectoralis major flap Groin flap	291,062	25,830	4 2 3 2	Neurolysis alone (10)	NR (24–108)
Killer and Hess^22^	Switzerland	Case series	12	13	4/8	NR (20–48)	RIBP (13)	Omental Flap	5	0	5	Conservative treatment (4) Neurolysis alone (4)	186 (60–348)
Minami et al.^23^	Japan	Case series	5	5	0/5	49.6 (29–72)	RIBP (5)	Latissimus dorsi flap	5	4	1	-	28 (25–39)
Millesi et al.^24^	Austria	Case series	9	9	3/6	39 (22–53)	RNTOS (5) RIBP (3) Post-osteosynthesis ACJ injury (1)	Adipofascial retropectoral flap	9	9	-	-	29.1 (13–61)
Sanders et al.^25^	USA	Case series	207	249	42/165	NR (13–64)	NTOS (185) RNTOS (64)	HA membrane	249	-	-	-	NR (12–24)
Patrick et al.^26^	Germany	Case report	2	2	1/1	52.5 (50–55)	RIBP (1) post-amputation axillary neuroma (1)	Serratus anterior flap	2	2	-	-	3 (3–3)
Annest and Sanders^18^	USA	Technical report + Case series	34	36	5/29	NR	RNTOS (36)	Latissimus dorsi flap	36	36	0	-	43 (6–84)
Sanders and Annest^27^	USA	Case report	1	1	0/1	35	RNTOS (1)	Amniotic membrane	1	-	-	-	3
Dolan et al.^28^	UK	Case series	16	16	12/14	39.6 (22–75)	RNTOS (15) RIBP (1)	Adipofascial deltopectoral flap	16	16	-	-	43.2 (25.2–126)
Oliveira et al.^29^	Brasil	Case report	1	1	0/1	68	RIBP (1)	Omental Flap	1	0	1	-	6
Jammeh et al.^17^	USA	Case series	90	90	25/65	39.9 (17–79)	RNTOS (90)	PLA film	90	-	-	-	67.2 (12–132)
Jammeh et al.^30^	USA	Case series	85	85	21/64	36.9 (15–64)	RNTOS (85)	PLA film	85	-	-	-	66 (16–133)
Teijink^31^	Netherlands	Technical report	19	19	NR	NR	NTOS (1) RNTOS (18)	Scalene fat pad divided in two layers	19	19	-	-	NR
Crawley et al.^32^	USA	Case series	1	1	0/1	22	RNTOS (1)	Amniotic membrane	1	-	-	-	24

NR, Not reported; RNTOS, Recurrent neurogenic thoracic outlet syndrome; RIBP, Radiation induced brachial plexopathy; ACJ, Acromioclavicular joint; HA, Hyaluronic acid; PLA, Polylactic acid.

### Surgical nerve wrapping techniques

**Figure 3:**
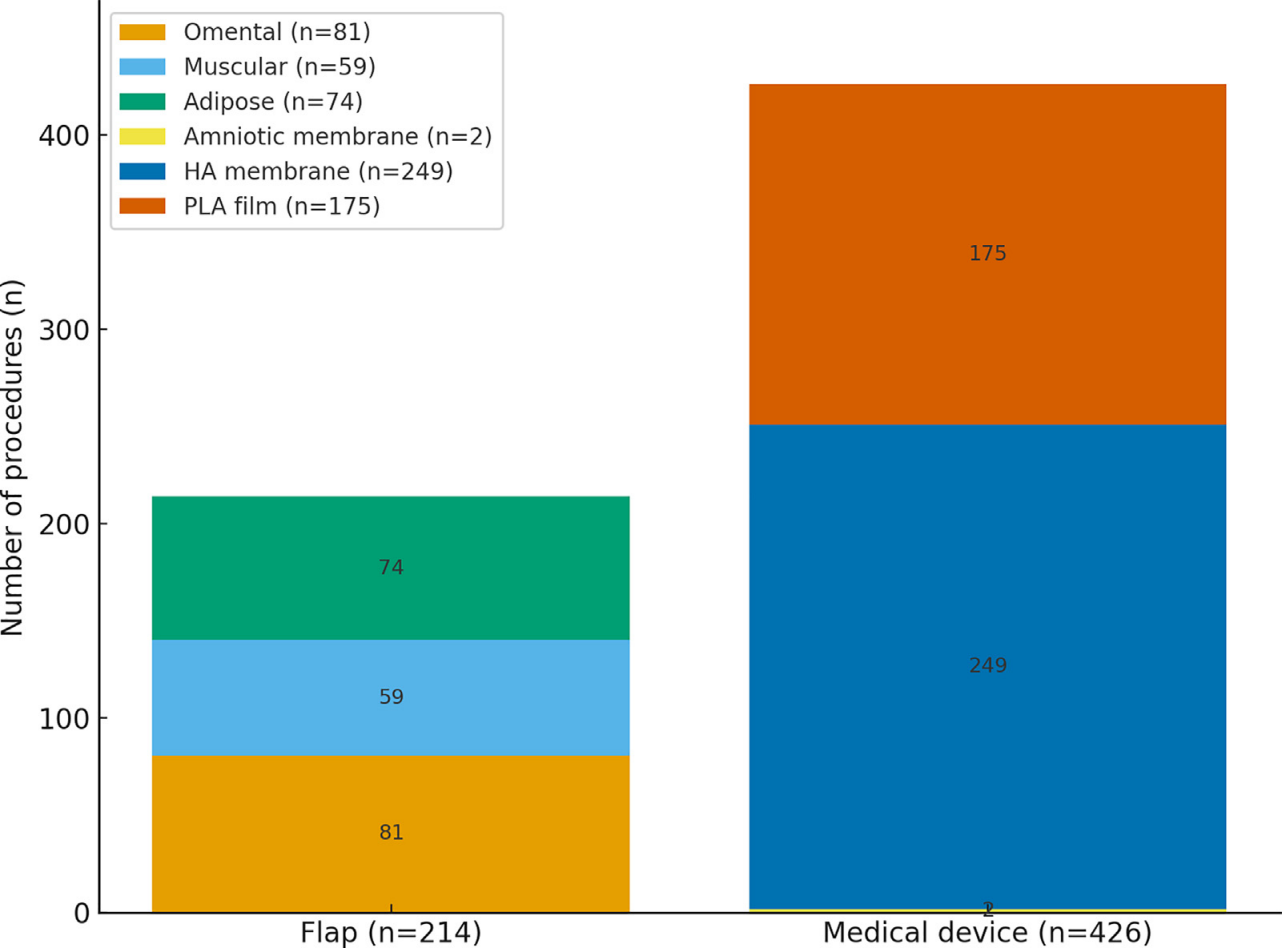
Number of procedures by tissue type.

Our analysis identified 11 distinct techniques for brachial plexus wrapping, divided into two main categories: tissue flaps and medical devices. Figure 3 illustrates the distribution of cases by tissue type.

Flaps were described in 11 studies, involving a total of 214 cases with significant tissue diversity. The greater omentum was the most frequently used flap (*n* = 81), followed by muscle flaps including the latissimus dorsi (*n* = 51), pectoralis major (*n* = 6), and serratus anterior (*n* = 2). Adipose tissue flaps accounted for 74 cases, including the scalene fat pad (*n* = 47), adipofascial deltopectoral flap (*n* = 16), retropectoral adipofascial flap (*n* = 9), and groin flap (*n* = 2).

The majority of flaps (75.2 %, *n* = 161) were pedicled, while 53 cases (24.8 %) used free flaps. The greater omentum, latissimus dorsi, and pectoralis major flaps were described in both pedicled and free forms, depending on the study. The groin flap was exclusively used as a free flap. Most flaps provided sufficient arc of rotation to cover the brachial plexus. However, authors such as Lê-Quang^21^ emphasized the interest of free flaps in specific situations: when the pedicle was too short to reach the most cranial part of the plexus, or in cases of significant tissue irradiation requiring contralateral harvesting, as with the latissimus dorsi or pectoralis major flaps.

In parallel, five studies reported the use of medical devices for 426 nerve wrapping procedures.^17^^,^^25^^,^^27^^,^^30^^,^^32^ These devices included amniotic membrane (*n* = 2), polylactic acid film (SurgiWrap®, MAST Biosurgery Inc., San Diego, CA, USA) (*n* = 175), and hyaluronic acid-based membrane (Seprafilm®, Baxter International Inc., Deerfield, IL, USA) (*n* = 249). SurgiWrap® is a bioabsorbable film designed to resorb within 3 to 4 months. Seprafilm® is a synthetic, translucent, implantable, and bioresorbable membrane composed of synthetic hyaluronic acid and carboxymethylcellulose, which transforms into a gel within 24 to 48 h after implantation and is fully resorbed within three to 4 weeks.

### Outcomes

The data collected showed marked heterogeneity both in the parameters assessed and in the measurement methods used. This variability, combined with the use of both self-reported (by patients) and observer-reported (by surgeons) outcomes, limits our analysis to a primarily descriptive approach. These data are presented in Table 3.

**Table 3:** Summary of postoperative outcomes.

Author (year)	*n*	Subgroups (*n*)	Pain	Motor / Sensory / Global Function	Scar / Donor site	Global assessment	Complications	Reoperations
Sessions^19^	37	-	NR	NR	NR	Surgeon-reported outcome: Excellent 4/37 (10.8 %) Good 19/37 (51.4 %) Fair 4/37 (10.8 %) Poor 1/37 (2.7 %)	1 phrenic nerve palsy; 1 pneumonia; 1 hemopneumothorax; 1 lymphedema; 1 hematoma; 3 seromas; 1 Horner’s syndrome; 1 surgical site infection	0
Narakas^20^	40	Conservative treatment (11) Neurolysis alone (14) Omental flap (15)	Improvement 0/11 (0 %) Resolution 12/14 (85.7 %) Resolution 10/15 (66.7 %) Improvement 13/15 (86.7 %)	Sensory-motor improvement 0/11 (0 %) Motor improvement 3/14 (21.4 %); Sensory improvement 1/14 (7.1 %) Motor improvement 6/15 (40 %); Sensory improvement 2/15 (13.3 %)	NR	NR	1 spontaneous axillary artery rupture; 1 clavicular pseudarthrosis; 1 chronic osteitis; 1 relative ischemia of gastric fundus; 1 exposed clavicle bone	Omental flap 1 (7.1 %) High cervical cordotomy 1 (7.1 %) Local flap 1 (6.7 %)
Brunelli and Brunelli^16^	37	Neurolysis alone (6) Omental flap (31)	Resolution 0/6 (0 %) Improvement 4/6 (66.7 %) Resolution 28/31 (0 %) Improvement 31/31 (100 %)	Sensory-motor improvement 0/6 (0 %) Motor improvement 22/31 (71 %); ROM improvement 10/31 (32.3 %); Sensory improvement 22/31 (71 %)	Skin trophicity improvement 0/6 (0 %) Skin trophicity improvement 20/31 (64.5 %)	NR	2 wound dehiscences	Local flap 2 (5.4 %)
LeQuang^21^	57	Neurolysis alone (10) Omental flap (29) Muscular flap (16) Adipocutaneous flap (2)	NR	NR	NR	Surgeon-reported outcome : Symptom improvement 4/10 (40 %) Symptom improvement 7/29 (24.1 %) Symptom improvement 1/16 (6.3 %) Symptom improvement 1/2 (50 %)	12 lymphedemas	0
Killer and Hess^22^	13	Conservative treatment (4) Neurolysis alone (4) Omental flap (5)	Improvement 0/4 (0 %) Improvement 2/4 (50 %) Improvement 5/5 (100 %) Resolution 3/5 (60 %)	Sensory-motor improvement 0/4 (0 %) Sensory-motor improvement 0/4 (0 %) Sensory-motor improvement 0/5 (0 %)	NR	NR	NR	NR
Minami et al.^23^	5	-	Resolution 4/5 (80 %)	Motor improvement 3/5 (60 %); Sensory improvement 4/5 (80 %)	NR	NR	0	0
Millesi et al.^24^	9	RNTOS (5) RIBP (3) Post-osteosynthesis of ACJ injury (1)	Resolution 3/5 (60 %) Improvement 4/5 (80 %) Resolution 3/3 (100 %) Improvement 1/1 (100 %)	NR Motor improvement 0/2 (100 %) NR	NR	NR	0	0
Sanders et al.^25^	249	-	NR	NR	NR	Surgeon-reported outcome: Good to excellent 148/249 (59.4 %) Fair 34/249 (13.7 %) Poor 67/249 (26.9 %)	0	10 (4 %)
Patrick et al.^26^	2	RIBP (1) Axillary neuroma (1)	Improvement 1/1 (100 %) Improvement 1/1 (100 %)	NR	NR	NR	0	0
Annest and Sanders^18^	36	-	NR	NR	NR	Patient-reported outcome: Lost to follow-up 12/36 (33.3 %) Symptom improvement 23/24 (95.8 %); Mean symptom reduction: 60 %	1 postoperative anemia requiring transfusion; 1 surgical site infection; 1 pneumonia; 1 pulmonary atelectasis; 1 asthma exacerbation; 1 atrial fibrillation	0
Sanders and Annest^27^	1	-	Improvement 1/1 (100 %)	Strength and dexterity improvement 1/1 (100 %)	NR	Patient-reported outcome : Excellent 1/1 (100 %)	0	0
Dolan et al.^28^	16	RNTOS (15) RIBP (1)	Improvement 12/15 (80 %) Resolution 1/1 (100 %)	NR Motor improvement 0/1 (0 %)	Mean VSS score: 2.4 (0–4); 90 % of patients reported no deformity at donor site	Surgeon-reported outcome: Excellent 1/16 (6.2 %) Good 6/16 (37.5 %) Fair 5/16 (31.3 %) Poor 4/16 (25 %)	1 pneumothorax; 1 pneumonia; 1 epidermolysis; 2 infraclavicular hypoesthesias	0
Oliveira et al.^29^	1	-	Resolution 1/1 (100 %)	Sensory-motor improvement 0/1 (0 %)	NR	LENT-SOMA score improved from grade 4 to grade 2	0	0
Jammeh et al.^17^	90	-	NR	QuickDASH: 62.0 → 43.5 (−18.5)	NR	Patient-reported outcome: Excellent 9/90 (10 %) Good 32/90 (35.6 %) Fair 39/90 (43.3 %) Poor 10/90 (11.1 %)	0	7 (8 %)
Jammeh et al.^30^	85	-	NR	QuickDASH: 65.2 → 37.6 (−27.6)	NR	Patient-reported outcome: Excellent 20/85 (23.5 %) Good 36/85 (42.4 %) Fair 22/85 (25.9 %) Poor 7/85 (8.2 %)	0	11 (12.9 %)
Teijink^31^	19	-	NR	NR	NR	NR	NR	NR
Crawley et al.^32^	1	-	Resolution 1/1 (100 %)	Grip strength: 0 → 32 kg; Full range of motion; Resolution of paresthesias; QuickDASH: 98 → 11 (−87)	NR	NR	0	0

NR, Not reported; ROM, Range of motion; RNTOS, Recurrent neurogenic thoracic outlet syndrome; RIBP, Radiation induced brachial plexopathy; ACJ, Acromioclavicular joint; VSS, Vancouver scar scale; LENT-SOMA, Late effects normal tissue task force—subjective, objective, management, analytic scale (4 grades).

Pain assessment, the most frequently reported outcome (10 studies), was analyzed in 86 out of 640 patients (13.4 %). Complete resolution of pain was reported in 62.8 % of cases (*n* = 54), and significant improvement in 91.9 % of cases (*n* = 79). When analyzing results by indication, pain improvement was observed in 59 of 62 cases (95.2 %) of RIBP and in 18 of 22 cases (81.2 %) of NTOS. By comparison, neurolysis alone for RIBP resulted in pain improvement in 75 % of cases (18/24), while conservative management (without surgery) provided no benefit (0/15).

Neurological deficits were also assessed. Following nerve wrapping for RIBP, motor improvement was reported in 52.5 % of cases (31/59) and sensory improvement in 50 % (28/56). Only the study by Brunelli and Brunelli^16^ specifically addressed skin trophicity improvement, reporting regression of radiation-induced dermatitis in 64.5 % of cases (20/31) after greater omentum wrapping.

For RNTOS, three studies reported functional outcomes using the QuickDASH score to assess global upper limb function. They showed an average reduction of −23.3 points across 176 patients. These results exclusively concerned nerve wrapping using medical devices (PLA or amniotic membrane).

Furthermore, the study by Dolan et al.^28^ investigated donor site morbidity after harvesting of an adipofascial deltopectoral flap, noting good scar quality (mean Vancouver Scar Scale score of 2.4) and preservation of thoracic contour (anterior chest wall) in 90 % of cases.

Regarding the overall surgical outcome, generally defined as improvement in symptoms reported by the patient or observed by the surgeon, six studies graded outcomes from poor to excellent. Among 478 patients who underwent nerve wrapping, 276 (57.7 %) were classified as having good or excellent outcomes. In virtually all patients (477/478), the indication was either NTOS or RNTOS; outcomes for these two indications were not reported separately.^25^

Three other studies^18^^,^^21^^,^^29^ simply described global symptomatic improvement in 45.8 % of cases (33/72), with wide variation depending on the indication: 95.8 % improvement for NTOS (23/24) compared to 20.8 % for RIBP (10/48).

### Complications

Only two studies did not detail complications.^22^^,^^31^ Of the 15 studies analyzed, covering 666 procedures, six studies reported complications in 40 patients, corresponding to an overall complication rate of 6 %. These included skin complications (*n* = 4), notably three wound dehiscences requiring local skin flap coverage; neurological complications (*n* = 4), including phrenic nerve palsy and Horner's syndrome; respiratory complications (*n* = 7), generally related to intraoperative pleural breaches; lymphatic complications (*n* = 16), including 13 cases of lymphedema and three seromas; bone complications (*n* = 2), with clavicle pseudarthrosis and chronic osteitis in irradiated fields; vascular or hemorrhagic complications (*n* = 5), including spontaneous axillary artery rupture and relative ischemia of the gastric fundus following greater omentum flap harvesting, although detailed consequences were not reported; and surgical site infections (*n* = 2).

Regarding reoperations, 5.4 % (28/519) of patients who underwent surgery for NTOS or RNTOS required reintervention for symptom recurrence. Two patients who underwent neurolysis alone for RIBP required reoperation: one underwent plexus wrapping with a greater omentum flap, and the other underwent high cervical cordotomy for pain management.

## Discussion

Our systematic review highlights the limited number of studies addressing nerve wrapping techniques for the treatment of brachial plexopathies in conjunction with neurolysis. All included studies are retrospective and provide a low level of evidence. Outcome assessment criteria are heterogeneous and often non-standardized across studies, making meaningful comparisons difficult. For instance, methods for assessing pain, function, or overall outcomes vary considerably, preventing any rigorous meta-analysis. Consequently, the overall methodological quality of existing studies is considered low. Using the MINORS score, we identified a high risk of bias in almost all included studies. Furthermore, these studies encompass a wide range of underlying etiologies with distinct pathophysiological mechanisms, which limits the translatability of clinical outcomes from one study to another.

These limitations in the current literature underscore the need for higher-quality data and support the development of prospective, potentially randomized, studies. Nevertheless, this review provides an overview of currently available surgical techniques for brachial plexus wrapping.

### Brachial plexus wrapping options: flaps vs. biomaterial covers

The different surgical strategies identified in our literature review can be grouped into three categories:

**Non-vascularized synthetic materials:** PLA films (SurgiWrap®)^17^^,^^30^ and hyaluronic acid-based membranes (Seprafilm®)^25^ are bioabsorbable materials that have been proposed for brachial plexus wrapping in NTOS or RNTOS surgery. Sanders et al.^27^ reported abandoning the use of Seprafilm®, which did not appear to reduce recurrences compared to a historical cohort from the same team prior to its use. However, this film seemed to facilitate dissection during revision surgeries. Their team has now replaced Seprafilm® with a human amniotic membrane. The use of a PTFE membrane, such as Gore-Tex®,was also suggested by Sessions in 1989^12^, but we could not access the full text of this study, and the use of this material for nerve wrapping appears to have fallen into disuse.

These synthetic materials, while potentially providing a physical barrier to prevent adhesions with surrounding tissues, generally have little biological effect, particularly regarding modulation of local inflammation or neovascularization.

**Non-vascularized biological materials:** Nerve wrapping can also be achieved using acellular or autologous biological tissues, which act as an insulating sheath around the nerves without providing new vascularization. Our review reports the use of human amniotic membrane for brachial plexus wrapping in RNTOS by Sanders and Annest in 2018^27^ and Crawley in 2024.^32^ The amniotic membrane is a natural biological matrix rich in collagen and growth factors, known for its anti-adhesive, anti-fibrotic, and anti-inflammatory properties. Animal studies have demonstrated its ability to inhibit fibroblast proliferation and scar neovascularization, thus reducing perineural adhesions.^33^, ^34^, ^35^ In clinical practice, cases of ulnar nerve neurolysis at the elbow (for recurrent compression) and radial nerve neurolysis at the wrist (recurrent Wartenberg syndrome) with amniotic membrane wrapping have been described, resulting in significant pain relief and functional improvement.^36^^,^^37^

Regarding RNTOS, Sanders and Annest initially published the case of a patient undergoing revision surgery 1 year after supraclavicular brachial plexus decompression with application of an amniotic membrane due to symptom recurrence. During reoperation, they observed that the portion of the plexus covered by the amniotic membrane was free of adhesions, while the proximal, unprotected portion was encased in dense fibrous tissue, presumably responsible for the recurrent symptoms.^27^ The same authors later reported in a 2021 publication on RNTOS management that they had operated on 97 patients using this technique, with a 5 % recurrence rate at 1 year. However, these results have not been officially published in detail and should be interpreted with caution.^38^

Other nerve wrapping techniques have been documented for peripheral nerves but were not identified in our systematic review for the brachial plexus. These include collagen matrix nerve wraps^6^ and autologous vein conduits.^8^ As these approaches have not been reported for the brachial plexus, their efficacy in RNTOS remains uncertain.

**Vascularized autologous tissues (flaps):** The use of vascularized flaps to cover the brachial plexus after neurolysis is a well-established approach. The rationale is to provide a well-vascularized, living barrier that protects the plexus from external compressions and limits scar adhesions with adjacent structures. Indeed, tissue hypoxia induced by repeated surgeries increases oxidative stress and cellular apoptosis, thereby exacerbating inflammatory and proliferative processes leading to fibrotic scar formation. Vascularized tissue transfer aims to counteract this local hypoxia and thus limit fibrous tissue development around nerves.^39^

Different types of flaps have been documented, including omentum, muscle, and adipofascial tissue, as highlighted in our review.

The greater omentum flap has been specifically described for coverage of RIBP, with excellent results in pain relief. It can be used as a pedicled or free flap, and its large surface area allows complete coverage of the plexus. The omentum is extremely vascularized and pliable, potentially making it more effective than muscle flaps in reducing perineural fibrosis. This is supported by Brunneli et al. in a 1988 murine study showing that perineural fibrosis was five times greater when the injured nerve was wrapped with muscle compared to omentum.^40^ However, omentum harvest is highly invasive, requiring laparotomy, and carries a non-negligible risk of hernia or abdominal wall weakness.^41^

Muscle flaps have been used for both RIBP coverage^21^^,^^23^ and brachial plexus wrapping in RNTOS.^18^ In our review, the latissimus dorsi is the most frequently reported flap for both indications, while pectoralis major and serratus anterior flaps are used more sporadically. These flaps can be used as pedicled or free flaps, but microvascular anastomoses are usually unnecessary when harvested from the ipsilateral side.

Nevertheless, these flaps require extensive dissection in an already painful area and involve sacrificing muscle, adding morbidity to an upper limb often already functionally impaired.^42^ For example, harvesting the serratus anterior carries a high risk of scapular winging,^43^ a complication that can also result from plexus and long thoracic nerve dissection alone, making this option less favorable in our opinion.

Adipofascial flaps, on the other hand, avoid these drawbacks. They can be harvested locally or distantly as free flaps. Moreover, adipose tissue has inherent anti-inflammatory properties, mediated by anti-inflammatory cytokines secreted by adipose-derived mesenchymal stem cells. These cells may also reduce nociceptive hypersensitivity and promote peripheral nerve regeneration.^44^, ^45^, ^46^ Four different adipofascial flaps are reported in our review:

The first is the prescalene fat pad flap, harvested through a supraclavicular approach. The prescalene fat is mobilized laterally to allow plexus decompression, then repositioned to separate the plexus from adjacent structures. Teijink^31^ proposed a technical modification by dividing this fat pad into deep and superficial layers to wrap both sides of the plexus. However, this fat pad is mainly vascularized by the transverse cervical and supraclavicular arteries, which are frequently sacrificed during plexus exposure,^19^ raising concerns about flap viability. Additionally, in revision surgeries, this fat pad is often fibrotic, making its use for plexus coverage unreliable.

The second is the adipofascial deltopectoral flap described by Dolan et al. in 2020.^28^ This flap is also harvested through a supraclavicular approach, relying on perforators from the internal mammary artery originating from the second and third intercostal spaces. The authors described a flap measuring 10 × 10 cm. This strategy appears promising, as it is relatively low-morbidity and avoids microvascular techniques. However, it requires extensive local dissection, which may exacerbate fibrosis, and the arc of rotation of this internal thoracic artery–based flap is relatively limited, allowing coverage of the primary trunks only under some tension and without enabling complete circumferential wrapping.

The third is the retropectoral adipofascial flap reported by Millesi et al.,^24^ vascularized by the pectoral branch of the thoracoacromial artery. It consists of harvesting the thin adipofascial layer between the pectoralis major and minor muscles, then tunneling it under the clavicle to cover the plexus in the supraclavicular region. The average size is 16.3 × 12.2 cm, with a rotation arc that can extend coverage up to the mandible. However, harvesting this flap often requires detaching the pectoralis major from its clavicular and humeral insertion, leading to the same concerns about extensive dissection and donor site morbidity.

The fourth option, described by Lê-Quang in 1989,^21^ involves groin adipofascial flaps, used to cover RIBP. These flaps, vascularized by the superficial circumflex iliac artery, are relatively low-morbidity, with scars typically hidden under clothing. Their surface area allows for complete plexus coverage. The main limitation is the often short and variable pedicle length, making distant microanastomoses difficult if local recipient vessels have been ligated during previous surgeries.

### Future perspectives

Our systematic review identified eight types of flaps currently documented for brachial plexus coverage. However, this list is not exhaustive, and other surgical options may be.

Several local flaps may hold promise for brachial plexus wrapping but are currently underutilized or undocumented in this specific indication. For instance, the transverse cervical artery perforator (TCAP) flap and the supraclavicular flap—both based on branches of the transverse cervical artery (TCA)—could theoretically provide effective local solutions due to their proximity and reliable vascularity.^47^^,^^48^ However, these vessels are often sacrificed during primary or revision surgeries to facilitate brachial plexus exposure, limiting the feasibility of these flaps in recurrent cases.

The classical supraclavicular approach typically involves ligation of the TCA, dissection of the scalene fat pad (separated from the internal jugular vein), its lateral displacement during the procedure, and final repositioning to protect nerves. However, the vascular supply of this fat flap is poorly documented in the literature, and its viability remains uncertain, particularly when split, as proposed by Teijink.^31^ Future anatomical studies should further characterize the vascularization of this flap after TCA ligation and assess whether the supraclavicular approach could be adapted to preserve this pedicle during primary procedures. Such a strategy might allow fat flap nerve wrapping at the time of the initial surgery (e.g., NTOS decompression), with the aim of reducing recurrence.

Distant pedicled flaps such as the parascapular flap (based on the descending branch of the circumflex scapular artery)^49^ and the thoracodorsal artery perforator (TDAP)^50^ flap represent viable alternatives. These flaps offer several advantages: they are consistently present, can be harvested with low donor-site morbidity, and their volume can be tailored to the defect size. Extended versions can reach up to 25 × 20 cm for the TDAP^51^ and 15 × 30 cm for the parascapular flap,^52^ potentially enabling full plexus coverage.

Nevertheless, transferring these flaps to the supraclavicular region requires dissection of a substantial subcutaneous tunnel, which is not without risks. This maneuver may injure vascular structures during flap passage and provoke additional local fibrosis—particularly problematic in previously operated, scarred tissue beds.

Free flaps with longer pedicles may help overcome the limitations associated with short-pedicle flaps such as the groin flap. In this regard, the anterolateral thigh (ALT)^53^ and deep inferior epigastric perforator (DIEP)^54^ flaps are attractive options. Their long vascular pedicles facilitate anastomoses even when local recipient vessels are compromised. However, the DIEP flap is not suitable for all patients, especially those with low abdominal skin laxity, such as thin or athletic individuals.

Another interesting option is the medial sural artery perforator (MSAP) flap, which provides a discreet donor-site scar on the posterior leg and offers a pedicle length ranging from 9 to 16 cm.^55^ Its main drawback lies in the systematic need for intramuscular dissection, which may increase operative time and technical difficulty.

Before clinical implementation, dedicated anatomical and feasibility studies are needed to assess their reliability, reproducibility, and risks.

Finally, a promising avenue may lie in combining vascularized flap coverage with medical devices such as amniotic membrane nerve wraps. This dual approach could leverage the anti-adhesive and anti-inflammatory effects of biological membranes while benefiting from the protective and pro-angiogenic environment provided by vascularized tissues. Such a strategy might offer synergistic effects on nerve healing and long-term symptom control, but currently lacks supporting evidence in the literature.

## Conclusion

This systematic review reveals that although various surgical techniques exist for brachial plexus nerve wrapping, there remains a lack of robust data to standardize the indications for this approach. The available results suggest potential improvements in pain and functional outcomes, particularly in cases of RIBP treated with omental flaps and in thoracic outlet syndrome managed with resorbable membranes. However, the methodological heterogeneity of the included studies, their retrospective design, and the high risk of bias significantly limit the strength of these conclusions.

Future research should prioritize prospective protocols comparing nerve wrapping to isolated neurolysis, with standardized outcome measures including pain, motor recovery, and quality of life. In the meantime, this review provides a comprehensive overview of available options, highlighting the importance of a tailored approach that considers the etiology of the plexopathy, the condition of local tissues, and the surgeon's experience.

## Funding

No funding was disclosed by the authors.

## Authors’ statements

F.T. and U.L. collected the data. F.T. drafted the manuscript. G.G. and U.L. supervised the project. T.L. and P.P. critically revised the manuscript. All authors approved the final version.

## Declaration of AI and AI-assisted technologies in the writing process

Statement: During the preparation of this work, the authors used ChatGPT (GPT-5, OpenAI) to translate parts of the manuscript from French to English. After using this tool, the authors reviewed and edited the content as needed and take(s) full responsibility for the content of the publication.

## Ethical approval

Not required.

## Declaration of competing interest

The authors, their immediate families, and any research foundations with which they are affiliated have not received any financial payments or other benefits from any commercial entity related to the subject of this article.
